# Incremental Value of Adding S100B to NSE for High-Specificity Rule-in of Poor Neurological Outcome After Out-of-Hospital Cardiac Arrest

**DOI:** 10.3390/jcm15083043

**Published:** 2026-04-16

**Authors:** Seokjae Hong, Seungho Lee, Jung Soo Park, Jin Hong Min, Changshin Kang, Byung Kook Lee

**Affiliations:** 1 Department of Emergency Medicine, College of Medicine, Chungnam National University, Daejeon 35015, Republic of Korea; seokjae8948@gmail.com (S.H.); leesh4169@cnuh.co.kr (S.L.); laphir@cnu.ac.kr (J.H.M.); rosc@cnu.ac.kr (C.K.); 2 Department of Emergency Medicine, Chungnam National University Hospital, Daejeon 35015, Republic of Korea; 3 Department of Emergency Medicine, Chonnam National University Medical School, Chonnam National University Hospital, Gwangju 61469, Republic of Korea; bbukkuk@hanmail.net

**Keywords:** out-of-hospital cardiac arrest, prognosis, biomarkers, neuron-specific enolase, S100 calcium-binding protein B

## Abstract

**Background**: We evaluated whether adding S100B to NSE improved discrimination or high-specificity rule-in of poor neurological outcome after out-of-hospital cardiac arrest (OHCA). **Methods**: In this single-center retrospective cohort study, comatose adult OHCA survivors treated with targeted temperature management had NSE and S100B measured at 0, 24, 48, and 72 h after return of spontaneous circulation. At each time point, we assessed NSE alone, S100B alone, and a logistic model combining both biomarkers in paired complete cases. Discrimination was assessed using the area under the receiver operating characteristic curve (AUC). Rule-in performance was evaluated using a timepoint-specific threshold that achieved 100% specificity in our cohort. Poor neurological outcome was defined as cerebral performance category 3–5 at 6 months. **Results**: Among 124 patients, 66 (53.2%) had poor outcomes. AUCs were similar between NSE alone and the combination across all time points (all *p* > 0.3). At 48 h, the combination ruled in 46/65 (70.8%) patients with poor outcome versus 36/65 (55.4%) with NSE alone, identifying 10 additional patients and a 15.4-percentage-point difference (95% confidence interval, −5.6 to 23.6). **Conclusions**: Adding S100B to NSE did not improve overall discrimination. The higher 48 h rule-in yield was estimated imprecisely and should be interpreted cautiously. Our findings require external validation before they can be translated to clinical settings.

## 1. Introduction

Hypoxic–ischemic brain injury (HIBI) is a leading determinant of neurological outcomes after out-of-hospital cardiac arrest (OHCA) [1,2]. In practice, “ruling in” poor neurological outcome is linked to high-stakes decisions, including family counselling, goal-of-care alignment, and allocation of intensive care resources; in some settings, it may also inform treatment-limitation discussions. Because a false-positive prediction can cause irreversible harm, conservative prognostication prioritizes minimizing false-positive classifications [3,4].

Current international guidelines endorse a multimodal prognostication strategy and recommend serial neuron-specific enolase (NSE) measurements within 72 h after return of spontaneous circulation (ROSC), with the strongest utility typically around 48–72 h [1,2,5]. While S100 calcium-binding protein B (S100B) has also been extensively studied [6,7,8], the 2025 ERC/ESICM guideline does not recommend the routine use of S100B for prognostication, citing limited incremental value beyond existing models, including NSE [1]. Importantly, prior evaluations have largely focused on global discrimination (e.g., area under the receiver operating characteristic curve [AUC]), which may not capture decision-relevant performance at a zero–false-positive operating point [9].

Neurological outcomes after OHCA are influenced by multiple factors beyond biomarker release, including arrest characteristics, no-flow and low-flow duration, initial rhythm, and post-arrest care such as temperature management [10]. Accordingly, biomarker-only analyses should be interpreted as indicating incremental biomarker value rather than as full clinical prognostic models. Therefore, our aim was not to develop a transportable multimodal prediction model but to determine whether S100B adds prognostic information beyond NSE at prespecified serial time points. We aimed to determine whether adding S100B to NSE improved AUC-based discrimination and high-specificity rule-in yield at 0, 24, 48, and 72 h after ROSC. Because thresholds selected to achieve 100% specificity are sample-dependent, these analyses were intended as hypothesis-generating rather than as ready-to-use clinical cut-offs.

## 2. Materials and Methods

### 2.1. Study Design and Population

This retrospective analysis used data from a prospectively maintained registry of adult comatose OHCA survivors treated with targeted temperature management at the Chungnam National University Hospital (Daejeon, Republic of Korea) between August 2019 and August 2025. The Institutional Review Board approved the study (CNUH-2026-01-063) and waived the requirement for informed consent due to the retrospective design and use of de-identified clinical data.

We included adult comatose OHCA survivors treated with targeted temperature management (TTM) who had serum NSE and S100B measured at one or more prespecified time points (0, 24, 48, 72 h after ROSC). Exclusion criteria comprised traumatic arrest, cardiac arrest due to a primary neurological cause (e.g., intracranial hemorrhage), and use of extracorporeal membrane oxygenation (ECMO).

According to institutional practice, withdrawal of life-sustaining treatment (WLST) was not performed during TTM. After completion of TTM and rewarming, subsequent management, including consideration of organ donation or WLST, was determined after clinical evaluation and discussion with patients’ families.

### 2.2. TTM Protocol

All patients included in this study underwent TTM. For 24 h, their temperature was maintained at either 33 or 36 °C using a feedback-controlled surface cooling system (Arctic Sun^®^ 5000; BD, Franklin Lakes, NJ, USA). Prior to March 2022, the institutional protocol uniformly set the target temperature at 33 °C; after that date, the attending physician determined the target temperature, selecting either 33 or 36 °C based on the patient’s hemodynamic stability and cardiac arrest etiology (cardiac vs. non-cardiac origin or shockable vs. non-shockable). Following the maintenance phase of TTM, patients were gradually rewarmed to 37 °C at a controlled rate of 0.25 °C/h. Throughout the TTM process, patients received sedative agents and neuromuscular blockade. Additionally, they were provided standard intensive care based on international guidelines for post-cardiac arrest management, adapted to the institutional protocol [1,2].

### 2.3. Clinical Variables

We extracted the following prespecified clinical variables from the registry and medical records when available: age, sex, witnessed arrest, bystander cardiopulmonary resuscitation, initial rhythm (shockable vs. non-shockable), presumed arrest etiology (cardiac vs. non-cardiac), low-flow time, Charlson Comorbidity Index score, and target temperature.

### 2.4. Biomarkers and Time Points

Serum NSE and S100B were measured at 0, 24, 48, and 72 h after ROSC according to institutional practice. “0 h” was defined as the first available post-ROSC serum sample. Analyses were performed separately at each time point using paired complete cases in which both NSE and S100B were available.

For the primary analysis, we evaluated NSE and S100B individually and then fitted a two-marker logistic regression model including only NSE and S100B. We selected this biomarker-only primary model because we aimed to assess the incremental value of S100B beyond NSE, and the available sample size was insufficient to support full, stable multimodal model development at each time point. The predicted probability from this model was used as the “NSE–S100B combination” predictor for ROC and rule-in analyses.

All samples were analyzed at a single central laboratory, Green Cross Laboratories (GC Labs; Yongin, Gyeonggi-do, Republic of Korea). NSE and S100B were measured using electrochemiluminescence immunoassays (ECLIAs) with Elecsys NSE^®^ on the cobas e801 analyzer (Roche Diagnostics, Rotkreuz, Switzerland) and Elecsys S100^®^ on the cobas e411 analyzer (Roche Diagnostics, Mannheim, Germany), respectively. The analytical measurement ranges were 0.1–300 ng/mL for NSE and 0.005–30 μg/L for S100B. Because high-specificity operating points may vary across assay platforms and laboratory settings, the thresholds observed in this study should not be interpreted as generalizable clinical cut-offs.

### 2.5. Neurological Outcomes

Neurological outcomes were assessed 6 months after ROSC using the Glasgow–Pittsburgh Cerebral Performance Category (CPC) scale and were dichotomized as good (CPC 1–2) or poor (CPC 3–5).

### 2.6. Statistical Analysis

Categorical variables are presented as frequencies (percentages), and continuous variables as medians with interquartile ranges. Categorical variables were compared using the chi-square or Fisher’s exact test, and continuous variables using the Mann–Whitney U test.

Primary analyses were performed separately at 0, 24, 48, and 72 h after ROSC using paired complete cases. At each time point, we evaluated NSE alone, S100B alone, and the NSE–S100B combination. For the combination, a logistic regression model including NSE and S100B was fitted, and the predicted probability was used as the combined predictor. Discrimination for poor neurological outcome at 6 months was assessed using ROC curves and AUCs with 95% confidence intervals (CIs), and AUCs were compared using the DeLong test [11].

Rule-in performance was also evaluated using a timepoint-specific threshold that achieved 100% specificity in the corresponding paired sample. For each predictor and time point, this threshold was set just above the highest observed value among patients who achieved good outcomes. The established thresholds were cohort-specific and are thus not considered generalizable. Using this threshold, we report classification counts, sensitivity, specificity, and the number of poor-outcome patients ruled in. For comparisons between the combination and NSE alone, we additionally report the number of additional poor-outcome patients ruled in and the difference in sensitivity, with percentile-based 95% CIs obtained by nonparametric bootstrap resampling (5000 iterations). All tests were two-sided, and *p* < 0.05 was considered statistically significant. Analyses were performed using IBM SPSS Statistics version 27.0 (IBM Corp., Armonk, NY, USA), MedCalc version 22.023 (MedCalc Software Ltd., Ostend, Belgium), Python (statsmodels, scikit-learn, NumPy, and pandas), and R (glmnet, boot, and pROC).

## 3. Results

### 3.1. Patient Characteristics

**Figure 1:**
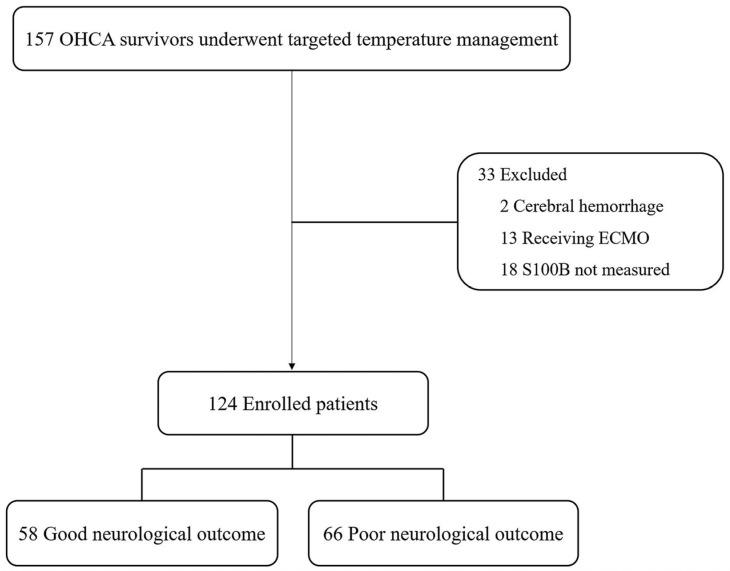
Flow diagram of the patients included in this study. Abbreviations: OHCA—out-of-hospital cardiac arrest; ECMO—extracorporeal membrane oxygenation; S100B—S100 calcium-binding protein B.

Among 157 OHCA survivors treated with TTM during the study period, 13 who underwent ECMO and 2 with cerebral hemorrhage were excluded. Of the remaining patients, 18 without available S100B measurements were excluded, leaving 124 patients for analysis. At 6 months after ROSC, 66/124 patients (53.2%) had poor neurological outcomes and 58/124 (46.8%) had good neurological outcomes (Figure 1). In the timepoint-specific paired analyses, 124, 116, 119, and 109 patients were included at 0, 24, 48, and 72 h after ROSC, respectively; the corresponding numbers of patients with poor neurological outcome were 66, 63, 65, and 57. Baseline characteristics according to neurological outcome are shown in Table 1. Patients with poor neurological outcomes were less likely to be male; to have witnessed arrest, bystander CPR, shockable rhythm, or cardiac etiology; and to have longer low-flow times. Moreover, a target temperature of 33 °C was more frequent in the poor-outcome group (all *p* < 0.05).

### 3.2. Timepoint-Specific Discrimination

**Figure 2:**
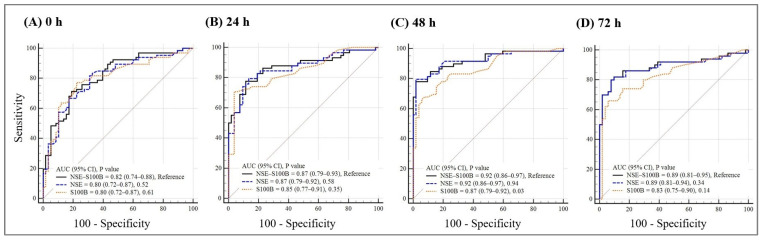
Timepoint-specific discrimination of NSE, S100B, and the NSE–S100B combination for poor neurological outcomes. Receiver operating characteristic curves compare neuron-specific enolase (NSE), S100 calcium-binding protein B (S100B), and the NSE–S100B combination for predicting poor neurological outcomes (CPC 3–5) at 6 months. Panels show biomarker performance at (**A**) 0, (**B**) 24, (**C**) 48, and (**D**) 72 h after return of spontaneous circulation (ROSC). Areas under the curve (AUCs) with 95% confidence intervals (CIs) are displayed in each panel. *p* values shown in the panels refer to comparisons between NSE alone and the NSE–S100B combination using the DeLong test.

AUCs for NSE alone and the NSE–S100B combination were similar at all time points (Table 2 and Figure 2): 0 h, 0.80 vs. 0.82 (*p* = 0.52); 24 h, 0.87 vs. 0.87 (*p* = 0.58); 48 h, 0.92 vs. 0.92 (*p* = 0.84); and 72 h, 0.89 vs. 0.89 (*p* = 0.34). Adding S100B to NSE did not improve overall discrimination at any evaluated time point.

### 3.3. Timepoint-Specific Rule-in Findings

Using the threshold with 100% specificity at each time point, the NSE–S100B combination ruled in poor outcome in 13/66 (19.7%) patients at 0 h, 33/63 (52.4%) at 24 h, 46/65 (70.8%) at 48 h, and 29/57 (50.9%) at 72 h. The corresponding values for NSE alone were 11/66 (16.7%), 29/63 (46.0%), 36/65 (55.4%), and 29/57 (50.9%), respectively (Table 3). The largest numerical difference was observed at 48 h; the combination identified 10 additional patients with poor outcomes compared with NSE alone.

### 3.4. Precision of Observed Rule-in Gain

**Figure 3:**
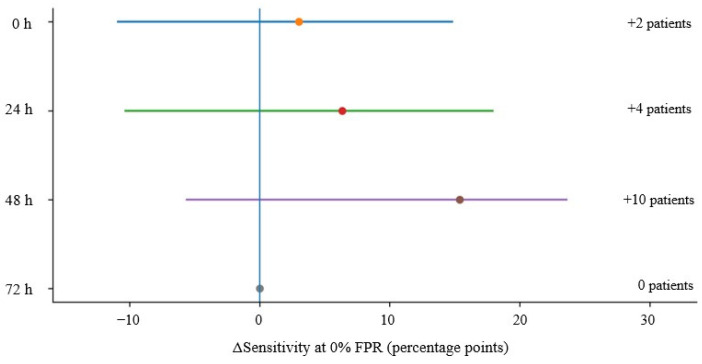
Difference in sensitivity between the NSE–S100B combination and NSE alone at a threshold with 100% specificity across time points. Points indicate the observed difference in sensitivity, and horizontal lines represent bootstrap 95% confidence intervals at 0, 24, 48, and 72 h after ROSC.

The observed difference in sensitivity between the NSE–S100B combination and NSE alone was 3.0 percentage points at 0 h (95% CI, −10.9 to 14.8), 6.4 percentage points at 24 h (95% CI, −9.8 to 17.9), 15.4 percentage points at 48 h (95% CI, −5.6 to 23.6), and 0.0 percentage points at 72 h (95% CI, 0.0 to 0.0). The corresponding numbers of additional poor-outcome patients ruled in were +2 (95% CI, −7 to +10), +4 (95% CI, −6 to +11), +10 (95% CI, −4 to +15), and 0 (95% CI, 0 to 0), respectively (Table 3 and Figure 3). Thus, the largest numerical gain was observed at 48 h, although the confidence interval for the change in sensitivity crossed zero.

## 4. Discussion

In this single-center cohort of comatose OHCA survivors treated with TTM, adding S100B to NSE did not improve overall discrimination for poor neurological outcome at any evaluated time point. However, when rule-in performance was evaluated using a timepoint-specific threshold with 100% specificity, the largest numerical gain was observed at 48 h, where the combination identified 10 additional patients with poor outcome compared with NSE alone. However, because the corresponding confidence interval for the difference in sensitivity crossed zero, this observed increase should be interpreted cautiously.

In a large substudy of the TTM trial, S100B improved discrimination when added to a baseline clinical model but did not further improve AUC when added to the model with NSE [6]. Our findings are broadly consistent with that pattern. Although AUC-based comparisons were neutral, AUC is a global measure that summarizes the entire ROC curve and may be insensitive to differences confined to clinically relevant high-specificity regions [12]. For this reason, we complemented the AUC analysis with a high-specificity rule-in framework and reported the absolute number of additional poor-outcome patients identified beyond NSE alone. In post-cardiac arrest neuroprognostication, where falsely pessimistic predictions may have irreversible consequences, this operating point perspective may be clinically informative even when overall discrimination is unchanged [1,13,14].

The time-specific signal at 48 h may reflect complementary biological information captured by the two biomarkers. NSE is a marker of neuronal injury but is susceptible to hemolysis and assay-related variation [5,15]. S100B is largely astroglial and may reflect glial injury and blood–brain barrier disruption [6]. In our cohort, the incremental value of combining S100B with NSE appeared most evident at the intermediate time point of 48 h, whereas no additional rule-in gain was observed at 72 h. This pattern is compatible with time-dependent biomarker kinetics and suggests that any incremental value of S100B may be context- and time-specific rather than sustained across all later sampling points [6,16].

Current guidelines recommend multimodal neuroprognostication and being cautious against treatment-limitation decisions based on any single test [1,2]. Our study was not designed to develop a full integrated clinical prognostic model but rather to assess whether S100B provided incremental information beyond NSE at prespecified serial time points. In this context, S100B should be viewed, at most, as a potential adjunct to NSE rather than as a stand-alone prognostic marker [17].

This study has several limitations. First, the retrospective single-center design limits generalizability. Second, 18 patients were excluded because S100B measurements were unavailable, which may have introduced selection bias. Third, the thresholds used for high-specificity rule-in were derived within the same cohort in which performance was assessed and should therefore be interpreted as cohort-specific rather than as ready-to-use clinical cut-offs. Fourth, the observed 48 h increase in rule-in yield was imprecisely estimated, and the bootstrap confidence interval for the difference in sensitivity included zero. Fifth, neurological outcome after OHCA is influenced by multiple factors beyond biomarker levels, whereas this study was intentionally designed as a biomarker-focused analysis rather than a full multimodal prognostic model. In addition, the institutional TTM strategy changed during the study period, from a uniform 33 °C protocol to individualized selection of 33 or 36 °C, which may have introduced clinical heterogeneity not fully captured in this analysis [18]. Finally, patients receiving ECMO were excluded to reduce biomarker confounding, which may limit the applicability of our findings to clinical settings with frequent extracorporeal resuscitation. These findings should be validated in prospective multicenter studies.

## 5. Conclusions

Combining S100B with NSE did not improve AUC-based discrimination for poor neurological outcomes after OHCA. At 48 h, the combination showed a numerically higher rule-in yield than NSE alone when evaluated using a timepoint-specific threshold with 100% specificity, identifying 10 additional patients with poor outcomes; however, this effect was imprecisely estimated. These findings should be considered hypothesis-generating and require external validation before clinical application.

## Figures and Tables

**Table 1:** Baseline characteristics according to neurological outcome at 6 months.

Characteristics	Cohort (n = 124)	Good Neurological Outcome (n = 58)	Poor Neurological Outcome (n = 66)	*p*-Value ^a^
Age, years	60.0 (51.0–71.0)	60.0 (51.8–65.8)	60.0 (50.0–73.3)	0.34
Male sex	90 (73)	49 (85)	41 (62)	0.008
CCI score	3.0 (1.0–4.0)	2.0 (1.0–4.0)	3.0 (1.0–5.0)	0.16
Arrest characteristics				
Witnessed arrest	78 (63)	47 (81)	31 (47)	<0.001
Bystander CPR	78 (63)	42 (72)	36 (55)	0.04
Shockable rhythm	55 (44)	39 (67)	16 (24)	<0.001
Cardiac etiology	66 (53)	46 (79)	20 (30)	<0.001
Low-flow time, min	20.5 (12.0–33.3)	15.0 (9.0–22.0)	27.5 (18.8–39.0)	<0.001
Target temperature 33 °C, n (%)	82 (66)	27 (47)	55 (83)	<0.001

Continuous variables are presented as median (IQR); categorical variables as number (%). ^a^ *p* values were calculated using the χ^2^ test for categorical variables and the Mann–Whitney U test for continuous variables. Abbreviations: CCI—Charlson Comorbidity Index; CPR—cardiopulmonary resuscitation.

**Table 2:** Timepoint-specific discrimination of NSE, S100B, and the NSE–S100B combination.

Time After ROSC	N (Paired)	Poor Outcome (n)	AUC NSE (95% CI)	AUC S100B (95% CI)	AUC Combination (95% CI)	*p*-Value ^a^ (NSE vs. Combination)
0 h	124	66	0.80 (0.72–0.87)	0.80 (0.72–0.87)	0.82 (0.74–0.88)	0.52
24 h	116	63	0.87 (0.79–0.92)	0.85 (0.77–0.91)	0.87 (0.79–0.93)	0.58
48 h	119	65	0.92 (0.86–0.97)	0.87 (0.79–0.92)	0.92 (0.86–0.97)	0.84
72 h	109	57	0.89 (0.81–0.94)	0.83 (0.75–0.90)	0.89 (0.81–0.95)	0.34

^a^ *p* values were calculated, with AUCs compared using the DeLong test. Abbreviations: AUC—area under the receiver operating characteristic curve; CI—confidence interval; ROSC—return of spontaneous circulation; NSE—neuron-specific enolase; S100B—S100 calcium-binding protein B.

**Table 3:** High-specificity rule-in performance across time points.

Time After ROSC	N (Paired)	Poor Outcome (n)	NSE Ruled-in n/Npoor (%)	Combination Ruled-in n/Npoor (%)	Additional Ruled-in (n)	ΔRule-in (95% CI)	ΔSensitivity %*p* (95% CI)
0 h	124	66	11/66 (16.7%)	13/66 (19.7%)	+2	–7 to +10	3.0 (–10.9–14.8)
24 h	116	63	29/63 (46.0%)	33/63 (52.4%)	+4	–6 to +11	6.4 (–9.8–17.9)
48 h	119	65	36/65 (55.4%)	46/65 (70.8%)	+10	–4 to +15	15.4 (–5.6–23.6)
72 h	109	57	29/57 (50.9%)	29/57 (50.9%)	0	0 to 0	0.0 (0.0–0.0)

Percentages for ruled-in patients are calculated using the number of poor-outcome patients at each time point as the denominator. Rule-in thresholds were selected to achieve 100% specificity in the corresponding paired sample. Differences in rule-in counts and sensitivity were estimated by bootstrap resampling (5000 iterations). Abbreviations: CI—confidence interval; ROSC—return of spontaneous circulation; NSE—neuron-specific enolase.

## Data Availability

The data presented here are available on request from the corresponding author. The data are not publicly available due to ethical concerns.

## Abbreviations

The following abbreviations are used in this manuscript:

AUC	Area under the receiver operating characteristic curve
CI	Confidence interval
CPC	Cerebral performance category
ECMO	Extracorporeal membrane oxygenation
ERC	European Resuscitation Council
ESICM	European Society of Intensive Care Medicine
HIBI	Hypoxic–ischemic brain injury
IQR	Interquartile range
NSE	Neuron-specific enolase
OHCA	Out-of-hospital cardiac arrest
ROC	Receiver operating characteristic
ROSC	Return of spontaneous circulation
S100B	S100 calcium-binding protein B
TTM	Targeted temperature management
WLST	Withdrawal of life-sustaining treatment
